# Medial strabismus (esotropia) at rest associated with contralateral paramedian thalamic ischemic infarction in 2 dogs

**DOI:** 10.1111/jvim.16986

**Published:** 2024-01-11

**Authors:** Theofanis Liatis, Ioannis N. Plessas, Holger Volk, Danielle Whittaker

**Affiliations:** ^1^ Queen Mother Hospital for Animals, Royal Veterinary College University of London Hatfield UK; ^2^ Davies Veterinary Specialists Hitchin UK; ^3^ Department of Small Animal Medicine and Surgery University of Veterinary Medicine Hannover Hannover Germany

**Keywords:** cerebrovascular accident, motor nucleus of oculomotor nerve, paramedian thalamus, pseudoabducens palsy, thalamic esotropia

## Abstract

Pseudoabducens paralysis resulting in resting medial strabismus (esotropia) is a rare consequence of a contralateral paramedian thalamic ischemic infarction in people. To date, esotropia has been reported in dogs in association with ipsilateral abducens neuropathy or extraocular myopathy, but not secondary to thalamic lesions. A 7‐year‐old male neutered Border Collie and a 12‐year‐old female neutered cross‐breed dog were presented with peracute nonprogressive vestibular ataxia. Neurological examination identified right esotropia, nonambulatory tetraparesis, right head tilt, vestibular ataxia and nystagmus. Lesions in both dogs were localized to the vestibular system with thalamic involvement. Magnetic resonance imaging of the brain identified a left paramedian thalamic lacunar ischemic infarct in both dogs. Interruption of descending inhibitory pathways that decussate in the subthalamic region and innervate the contralateral motor nucleus of the oculomotor nerve leads to hypertonicity of the medial rectus. These cases indicate that esotropia is a rare but highly localizing sign in dogs with contralateral thalamic infarcts.

**Abbreviations**
ADC: apparent diffusion coefficient
DWI: diffusion‐weighted imaging
FLAIR: fluid attenuated inversion recovery

## 1 INTRODUCTION

Pseudoabducens paralysis or palsy refers to failure of ocular abduction that is not caused by dysfunction of the abducens nerve, but likely is a result of increased convergence activity to the eye.^1^ In humans, pseudoabducens paralysis is a rare neurological sign, and it has been described as ipsilateral or contralateral to an ischemic infarct at the midbrain‐thalamic junction or thalamic‐subthalamic region, respectively.^2^ A paramedian thalamic lesion can cause interruption of the descending inhibitory convergence pathways that traverse the paramedian thalamus and decussate in the subthalamic region to innervate the contralateral motor nucleus of the oculomotor nerve. Such a lesion results in tonic activation of the medial rectus muscle of the contralateral eye and therefore contralateral resting medial strabismus (esotropia).^2^, ^3^

In dogs, neuro‐ophthalmological signs secondary to thalamic and midbrain ischemic infarcts have been reported rarely, and these signs include positional nystagmus, ipsilateral or contralateral positional ventral or ventrolateral strabismus, anisocoria with ipsilateral mydriasis, Horner syndrome and pupillary light reflex deficits,^4^, ^5^ whereas convergence‐retraction nystagmus and Collier's sign can be manifested when the dorsal midbrain is affected.^6^, ^7^ To date, esotropia has been reported in dogs in association with ipsilateral abducens neuropathy,^8^, ^9^ extraocular myopathy^10^ and congenital abnormalities,^11^ but not secondary to thalamic lesions.

Herein we describe esotropia as a localizing neurological sign of contralateral paramedian thalamic ischemic infarction in 2 dogs.

## 2 CASES

### 2.1 Case 1

**FIGURE 1:**
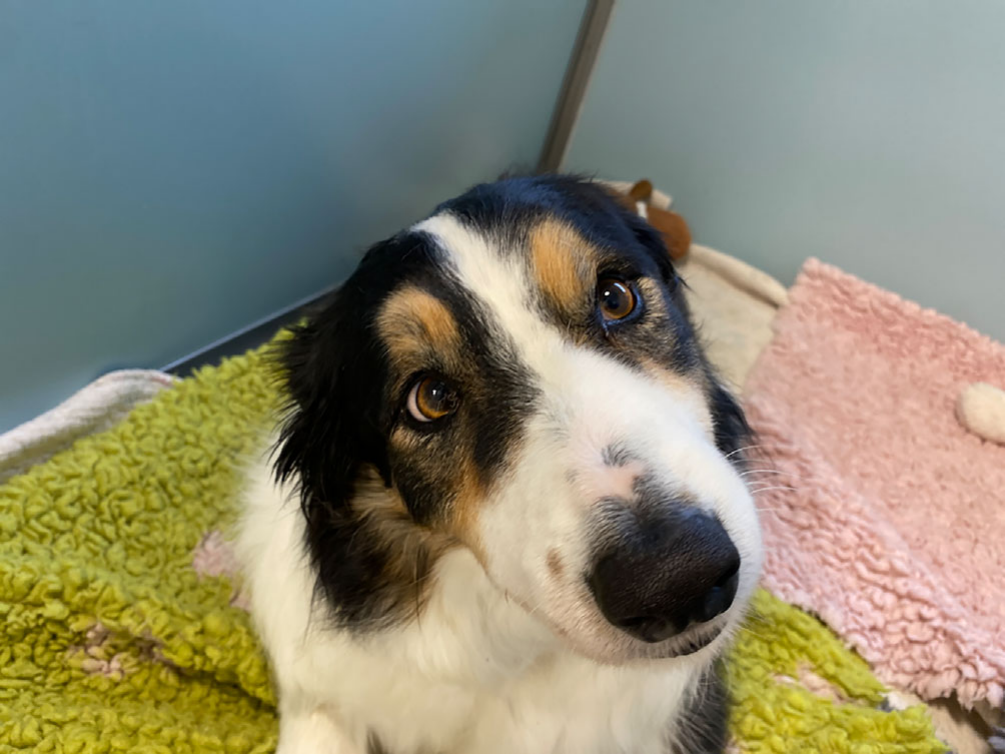
Photograph of the dog (case 1) diagnosed with a left paramedian thalamic ischemic infarct and a right contralateral resting medial strabismus (esotropia).

A 7‐year‐old male neutered Border Collie was presented to the hospital for evaluation of peracute nonprogressive vestibular ataxia. Physical examination was normal. Neurological examination identified marked right head tilt, nonambulatory tetraparesis and marked vestibular ataxia. The dog constantly rolled to the right side. Postural reactions could not be assessed. Cranial nerve assessment identified right esotropia (Figure 1), suspected upward gaze palsy with intact vestibulo‐ocular reflex. Episodic nonintentional head tremor with vertical direction from which the dog could be occasionally distracted also was present (Video 1). Neuroanatomical localization was consistent with a right‐sided central vestibular system lesion with suspected thalamic involvement based on the episodic head tremor.

**VIDEO 1:** Structural episodic nonintentional head tremor in a dog with a left paramedian thalamic ischemic infarct.

**FIGURE 2:**
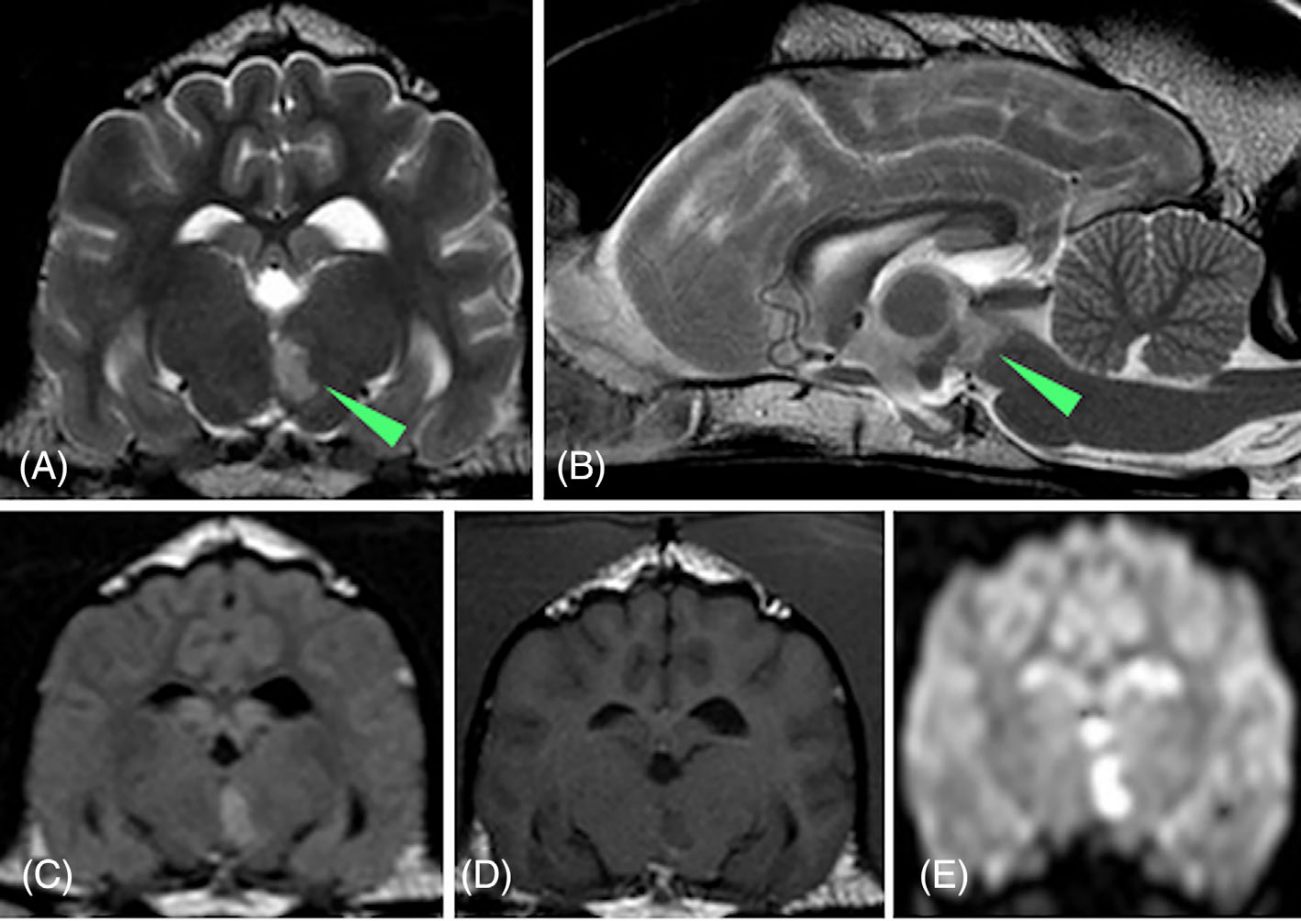
MRI of the head of case 1 including T2W transverse (A), T2W sagittal (B), T2 FLAIR transverse (C), T1W precontrast transverse (D), and DWI transverse (E) sequences, revealed a sharply‐marginated homogeneous lesion (arrowhead), that was T2W and T2W FLAIR hyperintense, T1W hypointense compared to the gray matter and noncontrast enhancing with restricted diffusion in DWI and ADC map in the left thalamus extending paramedian from the ventral level of the mesencephalic aqueduct to the ventral thalamus. This lesion was consistent with a left ventral lacunar paramedian thalamic ischemic infarct.

Venous blood gas analysis, hematology, thyroid profile (ie, total thyroxine and thyroid stimulating hormone concentrations), urinalysis and urine protein: creatinine ratio were within normal limits, whereas serum biochemistry disclosed mild hypercholesterolemia (8.7 mmol/L; reference interval [RI], 3.2‐6.2 mmol/L) and increased C‐reactive protein (37.7 mg/dL; RI, <10 mg/dL). Noninvasively obtained blood pressure (systolic arterial pressure using doppler) was considered normal (150‐160 mm Hg). Magnetic resonance imaging (MRI) identified a well‐marginated, intra‐axial lesion in the left thalamus extending paramedially from the ventral level of the mesencephalic aqueduct to the ventral thalamus. The lesion was T2W and T2 fluid‐attenuated inversion recovery (FLAIR) hyperintense, T1W hypointense compared to the gray matter, had no mass effect or contrast enhancement and showed restricted diffusion in diffuse weighting imaging (DWI) and apparent diffusion coefficient (ADC) mapping (Figure 2). This lesion was consistent with a left ventral lacunar paramedian thalamic ischemic infarct in the vascular territory of the caudal perforating artery.^5^ Computed tomography of thorax and abdomen disclosed no lesions. Thrombotic profile including viscoelastic coagulation monitoring was normal, whereas multiple electrode aggregometry analysis using arachidonic acid and adenosine diphosphate reagents suggested increased platelet activation and therefore a possible thrombotic event.

The dog was hospitalized for 7 days and given supportive care, including, IV fluid therapy, antinausea medications (maropitant, 1 mg/kg IV q24h; ondansetron, 1 mg/kg IV q12h) and physiotherapy. The dog subsequently improved to ambulatory status and was discharged from the hospital. The episodic head tremor completely resolved 9 days after the onset of the cerebrovascular accident.

### 2.2 Case 2

A 12‐year‐old female neutered cross‐breed dog was presented to the hospital for evaluation of peracute nonprogressive vestibular ataxia. Physical examination was normal. Neurological examination disclosed marked right head tilt, nonambulatory tetraparesis and marked vestibular ataxia. The dog rolled to the right side. Postural reaction assessment identified decreased hopping and paw placement in right thoracic and pelvic limbs. Cranial nerve assessment identified right esotropia and rotatory nystagmus (fast phase to the left). Neuroanatomical localization was consistent with a lesion in the right‐sided central vestibular system.

**FIGURE 3:**
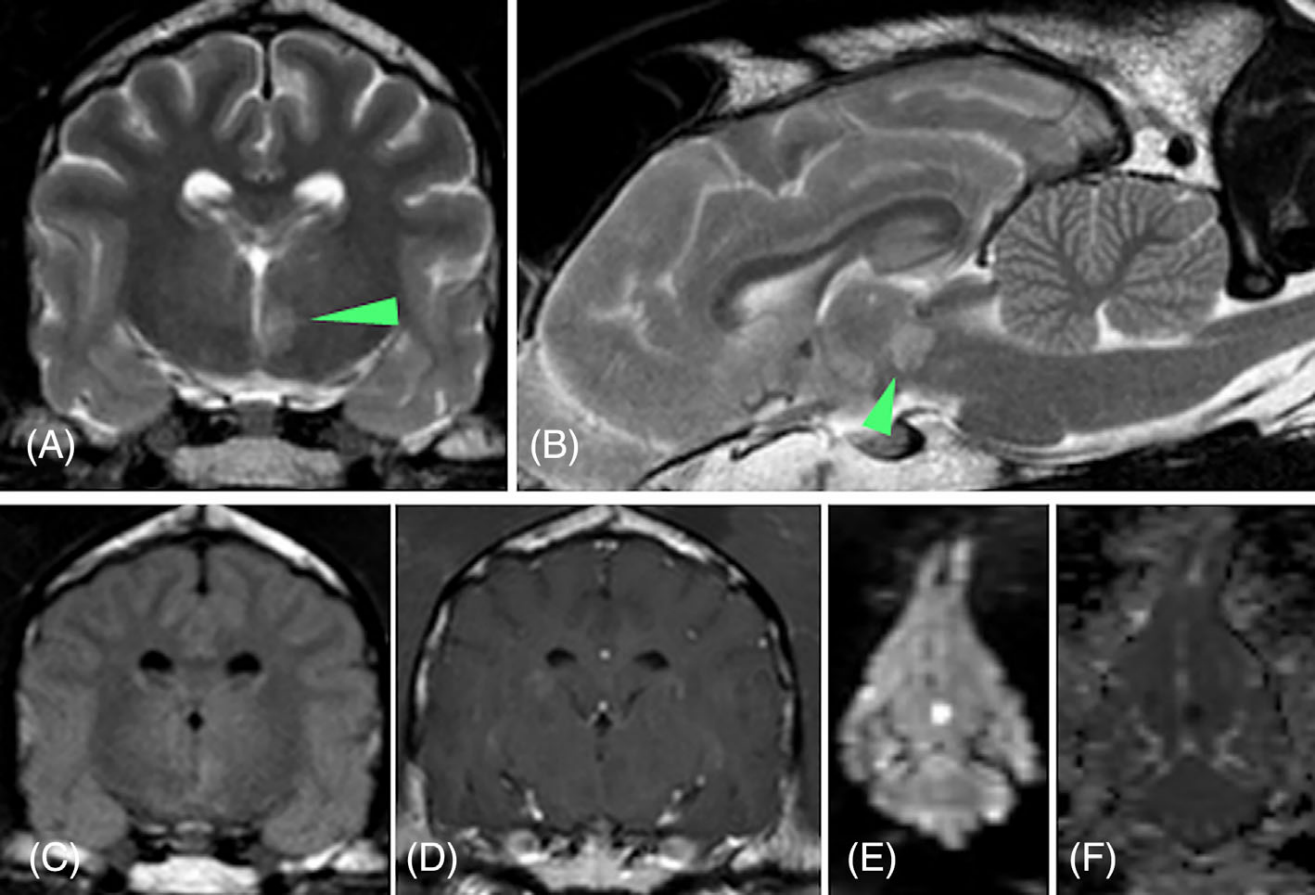
MRI of the head of case 1 including T2W transverse (A), T2W sagittal (B), T2 FLAIR transverse (C), T1W postcontrast transverse (D), DWI dorsal (E) and ADC map dorsal (F) sequences, revealed a sharply‐marginated, homogeneous lesion (arrowhead) that was T2W and T2 FLAIR hyperintense, T1W isointense compared to the gray matter, and noncontrast enhancing with restricted diffusion in DWI and ADC map lesion in the rostral aspect of the left thalamus. This lesion was consistent with a left ventral lacunar paramedian thalamic ischemic infarct.

Venous blood gas analysis, hematology, serum biochemistry and urinalysis findings were within normal limits. Noninvasivelly obtained blood pressure (systolic arterial pressure using doppler) was within normal limits (155 mm Hg). Magnetic resonance imaging identified a sharply and well‐marginated homogeneous lesion in the rostral aspect of the left thalamus, which was T2W and T2 FLAIR hyperintense, T1W isointense and noncontrast enhancing with evidence of restricted diffusion on DWI and ADC mapping (Figure 3). No mass effect was observed. This lesion was consistent with a left ventral lacunar paramedian thalamic ischemic infarct. Another lesion was observed in the left caudate nucleus with imaging features consistent with an old ischemic infarct. Antithrombin III activity was within normal limits.

The dog was hospitalized for 9 days and received IV fluid therapy, antithrombotics (clopidogrel 1.6 mg/kg PO q24h) and physiotherapy, subsequently improved to ambulatory status, and was discharged from the hospital.

## 3 DISCUSSION

Our findings suggest that esotropia is a rare but highly localizing neurological sign of a paramedian thalamic lesion, in these cases an ischemic infarct, in dogs.

In dogs, the oculomotor nerve is associated with a somatic motor nucleus and a parasympathetic nucleus (Edinger‐Westphal nucleus), both situated in the rostral midbrain.^12^ The motor nucleus consists of somatic efferent neurons that innervate the extraocular muscles of the eye (dorsal rectus, medial rectus, ventral rectus, and ventral oblique) and the *levator palpebrae superioris* muscle.^12^ The parasympathetic nucleus consists of visceral efferent neurons that innervate the ciliary ganglion (preganglionic axons) and, via the ciliary nerves, the ciliary muscle for lens accommodation and iris sphincter (dilator muscle of the pupil) for pupil constriction (postganglionic axons).^12^ The motor nucleus is located adjacent to the midline of the midbrain on the ventral border of the central gray matter, ventral to the mesencephalic aqueduct, whereas the parasympathetic nucleus of the oculomotor nerve lies rostral to the motor nucleus.^12^ Axons of the oculomotor nerve course ventrally through the reticular formation of the tegmentum. Some fibers cross to the opposite side but most remain ipsilateral, the actual proportions of which are not known for the dog.^12^ Axons from neurons of both somatic and visceral oculomotor nuclei join, running ventrally and exiting medial to the crus cerebri as oculomotor nerve roots. Additionally, there are also certain tegmental nuclei that are referred to as accessory oculomotor nuclei because of their proximity and functional relationship to the oculomotor nucleus (interstitial, prestitial, and precommissural nuclei).^12^

The extraocular muscles are the muscles responsible for the position and movement of the eye, and these are the medial rectus, lateral rectus, ventral rectus, dorsal rectus, dorsal oblique, ventral oblique, and retractor bulbi muscles.^12^ Strabismus refers to abnormal position of the eye caused by extraocular muscle disease, disease of the cranial nerves that innervate the extraocular muscles, or congenital malformation of the orbit.^12^ The oculomotor nerve innervates the ventral, dorsal and medial rectus muscles, and ventral oblique as well as the *levator palpebrae superioris*, and when affected by a lesion causes ventrolateral strabismus and ptosis, respectively.^12^ The trochlear nerve innervates the dorsal oblique muscle and lesions in it result in extorsional strabismus.^12^ The abducens nerve innervates the lateral rectus and the retractor bulbi muscles and lesions in it result in medial strabismus.^12^ Positional ventrolateral strabismus is observed in vestibular disease because of sensory input dysfunction.^12^ Rarely, central nervous system lesions have been associated with strabismus, including focal ischemic infarction in the midbrain, which has been described to cause ventrolateral strabismus in a dog.^12^

In humans, neurologic deficits to the extraocular muscles can be classified as supranuclear (or upper motor neuron), nuclear, and infranuclear (or low motor neuron) lesions.^13^ More specifically, any condition that disrupts signal transmission from the nucleus to the peripherally innervated region is considered infranuclear.^13^ Multiple supranuclear pathways travel from the cerebrum to the brainstem for different types of eye movements (ie, smooth pursuit, saccadic, vergence, and vestibulo‐ocular eye movements).^13^ These supranuclear pathways usually are independent of one another, and therefore when an eye movement is neurologically abnormal, neurologists focus on determining whether the deficit is caused by a supranuclear, nuclear or infranuclear lesion.^13^ An infranuclear and nuclear origin of the lesion can be ruled out clinically when an eye movement remains intact with 1 supranuclear pathway stimulation despite impairment by another.^13^

**FIGURE 4:**
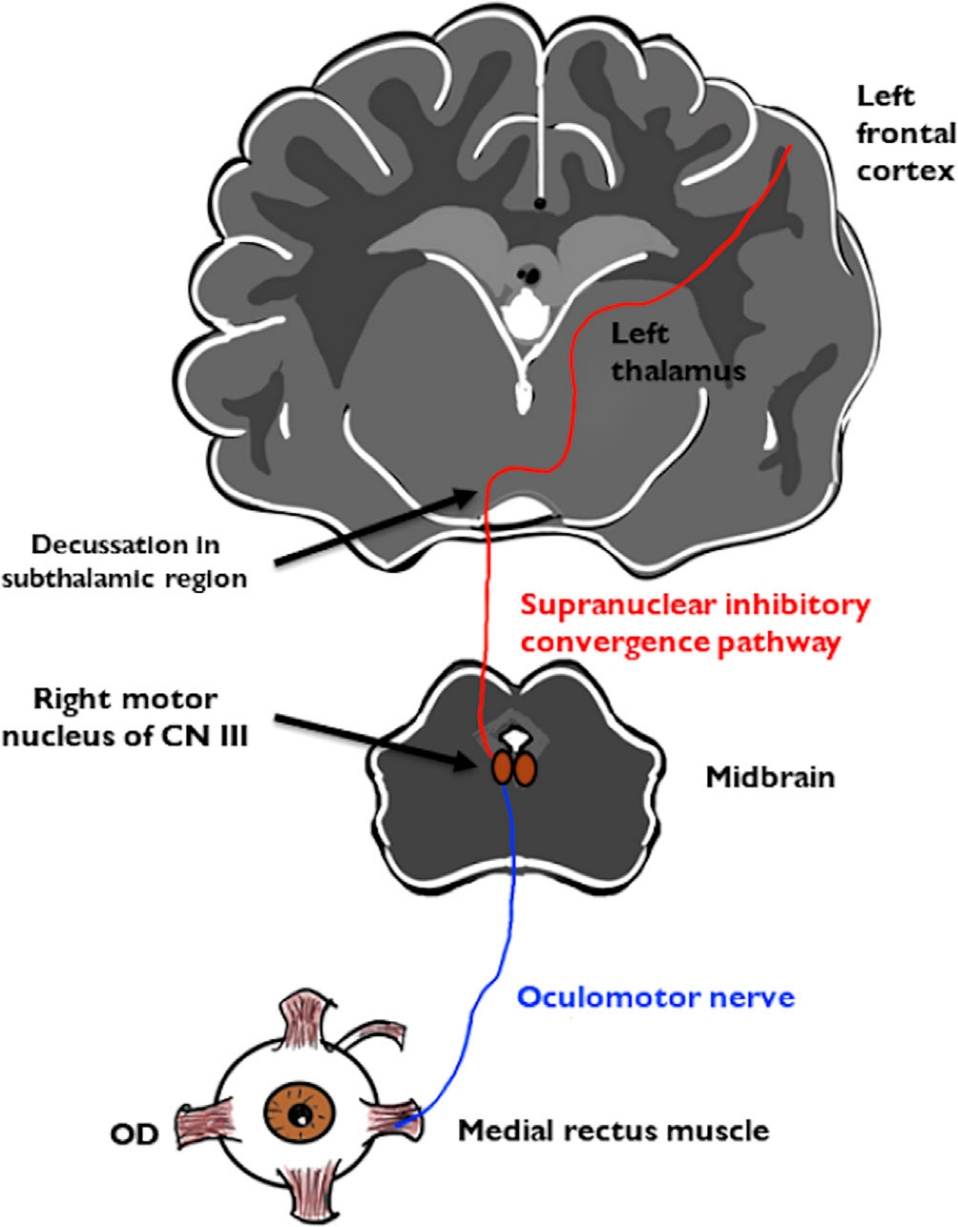
Schematic illustration of the disrupted pathways in dogs with pseudoabducens palsy.

When evaluating patients with ocular abduction deficits, lesions anywhere along the pathway from the extraocular muscles to the supranuclear areas that control horizontal saccades are considered for the motility deficit.^14^ Specifically, esotropia can be a result of a muscular, neuromuscular junction (eg, myasthenia gravis), abducens nerve (eg, anywhere along its course from the dorsal pons to the orbit) or supranuclear (eg, convergence spasm or increased convergence tone, divergence insufficiency) cause.^14^ Thalamic ischemic infarcts rarely result in neuro‐ophthalmological signs in humans. Although pseudoabducens palsy is rare, impairment of vertical gaze, vertical diplopia, skewed deviation or oculomotor nerve paralysis are more common.^3^ Pseudoabducens palsy is a neuro‐ophthalmologic sign occurring only in 5% of human patients with infarcts in the diencephalon.^15^ The exact pathophysiologic mechanisms of pseudoabducens palsy are unknown. Pseudoabducens palsy has been associated with lesions in the midbrain‐diencephalic region along with upward gaze palsy and convergence‐retraction nystagmus caused by dysfunction of convergence pathways.^2^ Nevertheless, pseudoabducens palsy also has been associated with solitary thalamic infarctions without midbrain involvement,^16^ in some of which upward gaze palsy is absent.^3^, ^16^ In humans, embolism of the paramedian artery, which supplies the intralaminal nuclear group and most of the dorsomedial thalamic nuclei of the thalamus, leads to infarctions in the thalamus and manifestation of pseudoabducens palsy.^3^ These nuclei send projections to the frontal eye field and supplementary eye field in the motor cortex and therefore, lesions in those nuclei could participate in the eye movement abnormalities.^3^ The subthalamus is proposed to be the region of decussation of these pathways.^2^ Therefore, it has been speculated that interruption of inhibitory convergence pathways as they transverse the paramedian thalamus leads to contralateral esotropia.^2^, ^3^, ^16^ This interruption consists of damage to direct inhibitory projections from the thalamus or impairment of input to midbrain neurons involved in the control of vergence eye movement.^3^ As a result, tonic activation of the medial rectus muscle pulls the eye medially causing contralateral esotropia (Figure 4).^3^

Vertical gaze palsy, which is defined as conjugate bilateral limitation of the eye movements in upgaze or downgaze, also can be seen in paramedian thalamic ischemic infarcts in humans.^17^ The rostral interstitial nuclei of the medial longitudinal fasciculus of the midbrain, which is located dorsal to the oculomotor nuclei, contain the final relays that produce all vertical saccades,^6^ and its dysfunction usually is associated with vertical gaze palsy.^17^ However, in paramedian thalamic lesions, vertical gaze palsy has been suspected to be associated with interruption of supranuclear fibers as they pass through the medial thalamus on their way to the control center in the midbrain.^17^ In these cases of supranuclear lesions, the vestibulo‐ocular reflex is expected to be intact.^17^ In our cases, vertical gaze palsy was suspected in 1 dog but its confirmation was challenging, whereas vestibulo‐ocular reflexes were intact. Additionally, our cases had a contralateral head tilt. Head tilt has been reported in dogs with contralateral paramedian thalamic lesions and it has been associated with dysfunction of the interstitial nucleus of Cajal or the medial longitudinal fasciculus of the midbrain.^5^ Finally, dog 1 manifested an episodic head tremor that had an acute onset along with the other neurological signs and ceased as soon as the dog improved neurologically. Structural episodic nonintentional head tremor in dogs has been associated with lesions in the thalamus, including the interthalamic adhesion and third ventricle, or mesencephalic aqueduct.^18^ Although its pathogenesis is unknown, it has been speculated that third ventricular cerebrospinal fluid flow imbalance or a compressive mass at that level might lead to an increase in the pressure effect and subsequent distortion of the dorsomedial red nucleus and dentatorubrothalamic pathways.^19^ In a recent study, there were no cases with a focal lesion such as a cerebrovascular accident.^18^ Our case indicates that a more specific region (ie, paramedian thalamus) might be associated with the manifestation of structural episodic nonintentional head tremor.

A paramedian thalamic ischemic infarct in dogs likely is attributed to thromboembolism of the caudal perforating artery arising from the basilar bifurcation and paramedian branches arising from the proximal portion of the caudal cerebral artery.^4^ In humans, the paramedian artery originating from the basilar artery is suggested to be involved in eye movement abnormalities of thalamic origin.^3^

Antithrombotic treatment is recommended when an ischemic infarct is suspected, and a concurrent medical condition associated with a risk of thrombosis is present based on current consensus.^20^ In case 1, which presented in 2023, no antithrombotic treatment was used in the absence of an underlying medical condition. In case 2, seen in 2011, bicavitary imaging to find a neoplastic cause or other source of embolization was not performed. Therefore, clopidogrel was given prophylactically for a potential unidentified source of the embolus. Additionally, a consensus statement on use of antithrombotic treatment was not available at that time.^20^

In conclusion, resting medial strabismus can be a localizing sign of contralateral paramedian thalamic ischemic infarction in dogs, and this condition might be associated with tonic activation of the medial rectus muscle because of disinhibition of the contralateral motor nucleus of the oculomotor nerve.

## CONFLICT OF INTEREST DECLARATION

Authors declare no conflict of interest.

## OFF‐LABEL ANTIMICROBIAL DECLARATION

Authors declare no off‐label use of antimicrobials.

## INSTITUTIONAL ANIMAL CARE AND USE COMMITTEE (IACUC) OR OTHER APPROVAL DECLARATION

Authors declare no IACUC or other approval was needed.

## HUMAN ETHICS APPROVAL DECLARATION

Authors declare human ethics approval was not needed for this study.
